# Barriers and facilitators to social prescribing in child and youth mental health: perspectives from the frontline

**DOI:** 10.1007/s00787-023-02257-x

**Published:** 2023-07-05

**Authors:** D. Hayes, A. Olsson, S. Begum, M. Bertotti, P. Jarvis-Beesley, E. Stapley

**Affiliations:** 1 Evidence Based Practice Unit (EBPU), University College London and the Anna Freud National Centre for Children and Families, London, England; 2 Research Department of Behavioural Science and Health, Institute of Epidemiology & Health Care, University College London, London, England; 3 The Healthcare Improvement Studies (THIS) Institute, University of Cambridge, Cambridge, England; 4 Institute for Connected Communities, University of East London, London, England; 5 Social Prescribing Youth Network (SPYN), Streetgames, Manchester, England

**Keywords:** Social prescribing, Theoretical domains framework, Child, Young person, Mental health, Well-being

## Abstract

There is growing interest in the role of Social Prescribing (SP) to help promote mental well-being and support individuals with mental health difficulties. Yet, implementation of SP to children and young people (CYP) has proved slow and underdeveloped compared with adult populations. Understanding the barriers and facilitators will help key stakeholders to better embed SP for CYP into practice. Using the Theoretical Domains Framework (TDF), a comprehensive, theoretical-led framework, underpinned by 33 behaviour change theories and 128 constructs, perceived barriers and facilitators to SP were investigated. The sample comprised of 11 Link Workers and 9 individuals involved in facilitating SP with CYP, who took part in semi-structured interviews. Transcripts were analysed using a deductive thematic analysis, and themes were coded under each theoretical domain. Overall, 33 barriers and facilitators for SP were identified across 12 domains of the TDF. Under capability, barriers and facilitators were found for knowledge, skills, memory/attention/decision making processes, and behavioural regulation. For opportunity, barriers and facilitators were found for social/professional influences, as well as environmental context and resources. Finally, for motivation, domains covered included: beliefs about consequences, beliefs about capabilities, optimism, motivations/goals, reinforcement, and emotions. Findings suggest that a wide range of barriers and facilitators affect the implementation of CYP SP to improve mental health and well-being. Interventions which target different domains related to capability, opportunity and motivation should be developed to better facilitate CYP SP.

**Supplementary Information:**

The online version contains supplementary material available at 10.1007/s00787-023-02257-x.

## Introduction

Social prescribing (SP) refers to the process of ‘enabling agencies and professionals to refer patients to an individual, often called a ‘link worker’, to co-design a non-clinical social prescription to improve their health and well-being’ [1, 2]. Also known by other names, such as ‘community referral’ [3], SP can encapsulate a wide range of social and community activities, including: community education groups, arts or creativity programmes, guided/health walks, volunteering, and supported education and employment [4]. Underpinned by evidence that socio-economic and cultural factors can have a greater impact on patient outcomes than healthcare interventions [5], SP works by tackling the wider determinants of health which can precipitate or perpetuate poor health and well-being [6, 7].

Internationally, there is increasing interest in SP, with countries across Europe [8–10], North America [11, 12] Australia [13], and Africa [14] rolling out SP initiatives. The emerging evidence points towards SP helping with a wide range of problems [4, 15]. For example, a review of SP interventions across different populations concluded that it leads to increases in well-being, overall mental health functioning and physical activity levels, as well as decreases in anxiety and depression [4]. Another review supports these findings, also suggesting other benefits such as increases in patient self-esteem and confidence [15]. Whilst promising, it has been noted that the quality of evidence remains low [4, 15] and that a number of service level barriers, including: leadership, management and organisational factors, staff turnover, staff engagement, as well as relationships and communication between partners and stakeholders, can affect successful and effective implementation of SP [16].

To explore the impact of SP on children and young people (CYP), a review was undertaken in 2020 [17]; however, no studies which focused on CYP were identified. One possible reason was that SP was too new with this population for the evidence to have permeated into the academic literature. However, an alternative possibility was that traditional SP pathways, which involves access via General Practitioners (GPs), may not be the preferred route for CYP to access SP. This latter point is supported by evidence, suggesting that CYP do not want to access mental health and well-being support via their GP due to short appointments and the belief that GPs would be of limited help [18]. As the majority of SP outcomes in the adult literature focus on mental health and well-being [4, 15, 19], this traditional pathway of SP may be acting as one barrier CYP accessing SP.

An updated review was conducted in 2022 [20], identifying four evaluations exploring SP for CYP mental health. Studies demonstrated that referral routes into SP were varied and included access via schools, child and adolescent mental health services (CAMHS), self-referral, as well as via GPs and primary care. Based on included studies, the review concluded that there was emerging evidence that SP improves mental health and well-being, particularly for older adolescents, as well as there being preliminary evidence to suggest a favourable return on investment [20].

As part of the review, factors affecting implementation were also explored and covered within two included studies [21, 22]. One study, which focused on vulnerable, at risk, teenage mothers, identified that interagency working proved difficult when implementing SP, with specific challenges around information sharing, as well as confusion around the referral processes [22]. Another study, a pilot of CYP SP across three sites in England, identified a number of challenges to implementation, including: referrals deemed inappropriate for SP, the need for greater co-ordination with agencies and parents/guardians, the sustainability of community assets and voluntary organisations that support SP, and the potential prohibitive cost and resource for CYP and families associated with participating in community activities [21].

Whilst initial barriers to the implementation of SP with CYP for mental health and well-being have been identified, they have, as of yet, not been systematically examined, and draw on only a few participants at select research pilot sites [21, 22]. This could result in limited learning, which may hamper the proposed rollout of SP for CYP [23]. The Theoretical Domains Framework (TDF) [24, 25] has been suggested as a tool to systematically explore barriers and facilitators to behaviours based on an individual or services capability, opportunity, or motivation, to do so. It has been previously used to explore implementing personalised care for young people [26, 27], as well as the barriers and facilitators to GPs implementing SP with adults [28]. In the latter example, barriers identified included: a lack of formal training around SP for GPs, little knowledge on community activities, the need for excellent interpersonal skills, the need for community groups in local areas, and the role of local primary care networks as well as commissioners influencing SP.

As SP gains momentum, both within the UK and abroad [8–13], and is rolled out to include CYP [14, 29, 30], a systematic exploration of the barriers and facilitators around its implementation is needed. Thus, the aim of this study is to explore barriers and facilitators to SP from the perspective of those working on the frontline with CYP to improve mental health and well-being.

### Research question

What are the perceived barriers and facilitators to SP with CYP who have mental health difficulties from the perspective of those on the frontline?

## Methods

### Recruitment and sampling strategy

During the 3-month sampling period, individuals and organisations working in SP with CYP were approached about the study. Three months was chosen due to study funding and associated timeframes to complete the project. At least fifteen individuals were deemed acceptable to allow for adequate information sufficiency [31] whilst bringing out different barriers that may affect SP. Adverts for the study were shared by email with relevant organisations and on social media. To increase sample diversity, the lead researcher (DH) and research assistants (AO and SB) targeted individuals working in SP at different organisations and locations across the UK (including major metropolitan areas and rural areas), aiming for variation in types of activities prescribed and the ages of CYP individuals worked with. The study was also promoted via the Social Prescribing Youth Network (SPYN), the Anna Freud National Centre for Children and Families (AFNCCF), the Emerging Minds Network, Public Health England, and the Child Outcomes Research Consortium (CORC), via social media and/or newsletters. Potential participants emailed the research team if they were interested in taking part.

### Participants

A total of 20 participants took part. This consisted of 11 Link Workers and 9 individuals involved in the setting up or running of SP for CYP. These wider roles included Link Worker Co-ordinators, SP Activity Co-ordinators, Professionals referring into SP services, and Managers and Heads of SP services and organisations.

The ages of Link Workers ranged from 23 to 45 (*M* = 29.81, SD = 7.77). Of the 11 Link Workers, 2 identified as male and 9 as female. Eight identified as ‘White or White British’, two as ‘Asian or Asian British’ and one as ‘Black or Black British’. Interviews lasted between 24.43 and 69.38 min (*M* = 42.21, SD = 13.89).

The ages of the individuals involved in the setting up or running of SP for CYP ranged from 25 to 60 (*M* = 43.11, SD = 11.75). Two identified as male and seven as female. All identified as ‘White or White British’. Interviews lasted between 21.21 and 77.54 min (*M* = 43.83, SD = 14.47).

### Procedure

This research follows the JARS–QUAL reporting guidelines for qualitative studies [32]. The lead and senior researchers were experienced in either qualitative methods, social prescribing and/or the TDF [24, 25] and research assistants were trained in qualitative methods and data analysis and had experience shadowing or conducting interviews. Researcher backgrounds included previous work in person-centred care, SP and community approaches to mental health and well-being with CYP. The creation of the interview schedule was developed in accordance with the TDF [24]. Thus, it was split into the three overarching categories on how behaviour can be influenced: capability, opportunity, and motivation. Under each overarching heading, interview questions were devised for each specific domain pertaining to how behaviour is influenced. Under capability there are four domains: knowledge, skills memory/attention/decision making, and behavioural regulation. Under opportunity there are two domains: environmental context and resource, as well as social influences. Whilst under motivation, there are seven domains: beliefs about capabilities, beliefs about consequences, optimism, intentions, goals, reinforcement and emotion.

Interview schedules were tested with a SPYN representative and a member of the research team who was also previously a social prescriber to ensure comprehension and clarity. Further changes following the first few interviews included minor clarifications to wording to ensure an understanding of key terms. The TDF was chosen as it explores the barriers and facilitators to behavioural phenomena, whilst being underpinned by theoretical constructs, helping to bridge the gap between theory and practice [24]. Moreover, its structured, but open-ended nature, was felt to help limit bias by stopping researchers focusing primarily on questions they deemed to be important, or participants focusing on barriers or facilitators that came to mind easily.

Following receipt of expressions of interest, the research team contacted the potential participant to discuss the study further and arrange a date and time for their interview over Microsoft Teams or by phone. Eleven participants declined to take part after expressing interest, with the most common reason being time commitments (64%). At the outset of the interviews, the researcher outlined that the research was part of a study exploring the barriers and facilitators to implementing CYP SP, specifically pertaining to mental health. Participants were then asked questions corresponding to the TDF. Participants could elaborate and deviate from script and prompts for specific domains were provided if needed (see interview schedule). The discussions were recorded using an encrypted Dictaphone, transcribed verbatim and anonymised after transcription, with identifying details (e.g., names of people and organisations) removed or changed. No follow-up interviews were conducted.

#### Data analysis

The transcribed interviews were analysed using thematic analysis [33] in NVivo [34]. Braun and Clarke [33] outline six steps that are undertaken as part of a thematic analysis. These consist of familiarising oneself with the data, the generation of codes, searching for themes, the reviewing of themes, defining and naming themes, and producing a report. A deductive approach was applied, which involved using existing overarching constructs (e.g., capability) and the specific theoretical domains in each (e.g., skills) derived from the Behaviour Change Wheel and TDF [24, 25] to code the data. However, there was scope to define subthemes inductively once coding under each domain was complete. The lead researcher (DH) developed a coding key in line with the TDF based on all transcribed interviews. This was then reviewed by the senior author (ES) to check that the codes reflected the content of the included data excerpts and transcripts. After this, the lead researcher (DH) refined the codes into subthemes, i.e., overarching categories encompassing all of the included codes. The senior researcher (ES) then checked that the codes and included data reflected each of the developed subthemes.

#### Ethical considerations

Ethical approval for this study was obtained from the University College London Research Ethics Committee (Project ID: 6386/002). During the consent process, participants provided explicit informed consent to be audio recorded and were reminded that they could pause or stop the interview at any time and that discussions were confidential unless disclosures of harm to the participant or other individuals were discussed. Participants received a £10 voucher as a thank you for participating. Audio recordings were deleted after transcript quality checking. Transcripts were anonymised.

## Results

Overall, 33 subthemes across twelve domains of the TDF [33] were identified as factors that were barriers or facilitators to SP for CYP with mental health difficulties. These spanned all three areas related to capability (see Table 1), opportunity (see Table 2) and motivation (see Table 3).

**Table 1:** Capability

Theoretical domain	Theme	Link workers (*n* = 11)	Those involved in running/managing SP schemes (*n* = 9)
1. Knowledge	A personalised understanding of each individual	✔	✔
	How to work with CYP	✔	✔
	Where the local community assets are and what they offer	✔	✔
	Policies and procedures specific to CYP SP	✔	✔
2. Skills	Interpersonal skills to engage CYP	✔	✔
	Networking skills to engage stakeholders	✔	✔
3. Memory, attention and decision-making	Deciding on who to involve and level of involvement	✔	✔
	Assessing risk and safety of CYP	✔	✔
	How to structure sessions	✔	
	Whether the referral is appropriate	✔	✔
	Evidencing effectiveness of SP		✔
4. Behavioural regulation	Monitoring service user feedback via questionnaires	✔	✔

**Table 2:** Opportunity

Theoretical domain	Theme	Link workers (*n* = 11)	Those involved in running/managing SP schemes (*n* = 9)
1. Environmental context and resource	Having appropriate spaces to talk to CYP	✔	✔
	Service limitations on who could be worked with	✔	✔
	Access to adequate clinical supervision	✔	✔
	Funds for CYP to engage in activities	✔	✔
	Tools to facilitate CYP engagement in SP	✔	✔
2. Social and professional influences	Clinical leadership involvement in SP design	✔	✔
	Community assets remit to accept or block onward referrals	✔	✔
	Policy and practice groups sway in the direction of SP		✔

**Table 3:** Motivation

Theoretical domain	Theme	Link workers (*n* = 11)	Those involved in running/managing SP schemes (*n* = 9)
3. Beliefs about capabilities	Feeling confident engaging with CYP	✔	
	Feeling less confident engaging with parents and guardians	✔	
	Feeling less confident that some clinicians saw a role for SP	✔	
	Feeling confident around implementing and executing SP		✔
4. Beliefs about consequences	Social prescribing empowers CYP and helps well-being	✔	✔
	CYP getting too attached	✔	
	Social prescribing is not a fix for everyone	✔	✔
5. Optimism	Social prescribing helps CYP	✔	✔
	The system is not working as well as it could be		✔
6. Motivation/Goals	Giving the CYP new opportunities and experiences	✔	✔
	Wanting to improving outcomes for CYP	✔	✔
7. Reinforcement	High job satisfaction from helping CYP	✔	✔
8. Emotion	Social prescribing can be draining and stressful	✔	

### Capability

Table 1 outlines twelve barriers and facilitators that were identified in relation to capability. These were found across four of the theoretical domains: knowledge, skills, memory/attention/decision-making, and behavioural regulation.

### Knowledge

#### A personalised understanding of each individual

The majority of Link Workers discussed that as part of SP, an understanding of the CYP was needed. This included their background and why they wanted to see a Link Worker:

*I want to know the demographics of the young person, and things like the age and if they’re in school…and see also what they want from a social prescriber (Link Worker 5)*

Such information was also highlighted as important by those involved in the setting up or running of SP, including SP Co-ordinators and Heads of Services:

*So knowing a bit of background about the child…why this might be appropriate for them to be referred to (Social Prescribing Co-ordinator 1)*

Link Workers highlighted that such information was sometimes difficult to gather, either because the CYP *“was reluctant to share information”* (Link Worker 5), or because the referring healthcare professionals did not provide it. When this occurred, Link Workers tried to ascertain this information from *“speaking to other professionals [or]…maybe looking through [the young person’s] medical record”* (Link Worker 7).

#### How to work with CYP

Both Link Workers and those involved in the setting up or running of SP for CYP highlighted that knowledge of how to work with CYP was essential to their roles.

*You're obviously going to need to know young people. I feel you need to have a good sense of young people* (Link Worker 11)

This included how to interact with CYP, a knowledge of the issues they face, and boundary setting:

*How to work with young people, how to put in boundaries, safeguarding …the kinds of things that they come up against* (Social Prescribing Manager 1)

Importantly, in organisations whose remit was broader than CYP, this knowledge also included an *“understanding of development, and developmental stages”* (Link Worker 3) and “*the differences between adults and children*” (Link Worker 7).

#### Where the local community assets are and what they offer

For successful SP to occur, a knowledge of local community assets was highlighted as important by both Link Workers and those involved in the setting up or running of SP:

*So I think one of the key things, the contacts in the community, knowing what's going on in the area so that you can link up with those services and those groups that are available* (Link Worker 1)

Link Workers and Co-ordinators often felt that they had a good knowledge of local assets, but acknowledged that the landscape could change quickly sometimes leaving them struggling to find other suitable sources of support.

#### Policies and procedures specific to CYP SP

Overall, both Link Workers and those involved in the setting up or running of SP for CYP highlighted that there were a lack of policies, procedures or guidelines relating to SP with CYP.

*So a lot of the information out there for social prescribing within the NHS is mainly for adults* (Link Worker 2)

What information was available was via a module provided by the SPYN. Consequently, Link Workers and those in management and implementation roles were limited to adapting resources which were already available for adults, as well as following policies and procedures embedded within their local organisation:

*Not really, not specific [guidelines] to children and young people. There are some guidelines for social prescribing with adults. For myself my background is paediatrics so I’ve kind of taken a lot of that, other guidance as kind of more generalised things and applied it* (Link Worker 4)

#### Skills

##### Interpersonal skills to engage CYP

All Link Workers and those involved in the setting up or running of SP for CYP highlighted the importance of interpersonal skills when working in a SP context. This included skills such as having *“empathy”* (Social Prescribing Co-ordinator 3), as well as skills around “*listening and encouraging*” (Social Prescribing Organisational Head 1). Most individuals felt that they had these skills via previously working with CYP:

*The need to enjoy working with people for a start, so they need to be happy to have those regular face-to-face or online conversations. And be interested and be able to listen, have empathy, and be non-judgemental, and to take an open view and be patient* (Social Prescribing Organisational Head 1)

##### Networking skills to engage stakeholders

For individuals involved in delivering SP, networking was seen as an important skill. It allowed for services to make others aware that SP was available within their area and that Link Workers were able to accept referrals:

*We need to know who to speak to, to promote our service, and who to connect with and network with, to ensure that we actually receive referrals and they know we’re here* (Social Prescribing Co-ordinator 1)

For others, networking also allowed for services to better understand the needs of their communities and what assets were out there:

*We network loads. We do know other organisations. We know our communities and their needs* (Social Prescribing Co-ordinator 2)

#### Memory, attention and decision-making

##### Deciding on who to involve and level of involvement

Link Workers outlined that one of the early decisions they made was how to involve CYP in SP. Providing that the CYP had capacity, some Link Workers spoke about supporting them to be involved in deciding activities, even if they thought others were a better ‘fit’:

*If an individual has capacity to make their own decision it’s important you allow them to make decisions that you wouldn’t necessarily deem as good* (Link Worker 1)

For other Link Workers and those involved in the setting up or running of SP for CYP, the involvement of family was also a consideration. This was particularly relevant when the CYP did not have capacity, or when the family structure was affecting the CYP’s mental health:

*As a children’s social prescriber I might be thinking about to what extent do I involve the family? How can we as a system help the family? Because kids are influenced by the context that they’re in.* (Social Prescribing Co-ordinator 3)

##### Assessing risk and safety of CYP

Link Workers and those involved in the setting up or running of SP for CYP stressed the importance of assessing risk and keeping the CYP safe. This pertained to both the mental health and well-being of the individual, but also the activities they may be participating in:

*The forefront of our work is safeguarding. We need to make sure these children are safe, they're obviously vulnerable and can be living in or involved in activities that perhaps are not the safest. So we do a lot of safeguarding and safety planning and protective behaviour work with our young people* (Social Prescribing Organisational Head 3)

Link Workers and those involved in the setting up or running of SP for CYP outlined that they understood how to pay attention to risk and implementing safeguarding protocols as necessary.

##### How to structure sessions

Another decision made by Link Workers was the structure of the sessions. Meetings with CYP would often be ‘CYP led’ and sessions paced according to what they felt comfortable sharing. Whilst on the surface the approach was flexible, Link Workers spoke about gently structuring sessions to facilitate movement towards the CYP’s goals:

*I would say that there isn’t really a clear beginning, middle and end in terms of their support. So yeah, there definitely has to be some form of decision making; obviously, they're in the driving seat, but in the background, you are sort of saying there is a structure to this, there is a destination; they can decide how they're going to get there, but you're definitely having in mind some sort of journey* (Link Worker 10)

Following on from conversations, Link Workers would then often go away and research available support for the CYP to present at the next session.

Another decision made by Link Workers was when to refer on and stop support.

*Also, which is a really big part that is overlooked, I think sometimes, is you need to decide when your support ends …such a hard part of social prescribing is ending it, because people get so attached, and it’s so hard to let people go and it’s so hard for them to let go of you* (Link Worker 9)

Given the time limited support around SP, some Link Workers felt ill equipped to manage endings. This was because they often formed close trusting relationships with CYP and did not feel equipped with the vocabulary and skills to smooth transitions.

##### Whether the referral is appropriate

When a referral came through, both Link Workers and SP Co-ordinators outlined that internal discussions were had around whether the referral was appropriate to take on:

*You need to decide whether that person is suitable or not for you. And then …you need to decide what you're going to do… where that person needs to go because you can’t just leave them in limbo* (Link Worker 9)

Decisions on what to do with the CYP included not accepting the referral, as well as picking the referral back up at a later point if it was felt more benefit could be added then:

*So, like, making sure that the referral is appropriate for me. Making sure that I can see them and add benefit to their situation. Sometimes there are lots of professionals already involved and I have to make an assessment of whether I can actually add something in a beneficial way at the current time. Or if maybe I need to step in later* (Link Worker 9)

The appropriateness of referrals was also echoed by SP Co-ordinators and Heads of Service, highlighting that referrals were discussed at team meetings, prior to accepting them onto an SP pathway:

*It’s something we have to consider every single time we receive a referral, we do work with children with very complex needs, and sometimes it’s either not safe or not appropriate for them to take part* (Social Prescribing Co-ordinator 1)

##### Evidencing effectiveness of SP

A couple of individuals involved in middle and senior management roles, outlined that part of their role remit was to think around how to evidence SP activity, which could be linked to funding key performance indicators:

*So I make decisions … about how we build the evidence base, so what research might we undertake to strengthen the business case and the evidence base for youth social prescribing* (Social Prescribing Organisational Head 1)

Linked with this, decisions about what metrics to use and how to demonstrate service need was discussed:

*Stuff around monitoring and evaluations. So how we evaluate the impact on young people. So that sits with me and I would work with the young people connectors to understand that and to make sure it’s being used and capturing what we need* (Social Prescribing Manager 1)

#### Behavioural regulation

##### Monitoring service user feedback via questionnaires

All Link Workers and those in management and implementation roles outlined that the impact of SP was monitored via questionnaires. Routinely used metrics included the ONS4 and the Warwick–Edinburgh Mental Well-being Scales:

*So my service as a whole uses the ONS4* (Link Worker 4)

Despite using these measures, some individuals felt that they were not appropriate to use with CYP as concepts were often not clear:

*Yeah. I think with the ONS4, it can be quite difficult because the questions are quite vague, and people generally, tend not to really know how to answer them* (Link Worker 6)

Others felt that these questionnaires did not get at the heart of what they were trying to achieve with SP. Therefore, they chose sometimes not to complete them with CYP:

*It’s quite difficult to measure. I think I’m meant to be doing like the Warwick-Edinburgh Scale and stuff like that. But I don’t. Terrible. But what I do see instead is those lifestyle changes and changes in attitude and in the things that they [young people] say* (Link Worker 7)

As a result, it was suggested by some participants that a more inclusive and user-friendly well-being measurement framework was needed, incorporating domains relevant and important to the CYP specifically:

*I think just tailoring it to things like a young person specifically would be experiencing, like with their family life or with their friends, or I guess with school as well* (Link Worker 5)

Alongside standardised metrics, the use of goal-based outcome measures were often used and preferred, due to their personalised, ideographic nature:

*I’ve devised a different outcome measure where we set some wellbeing goals and we score on a scale of 1 to 10 and it’s also got an emoji face underneath each number. So they can grade kind of how important this goal is to them, how kind of close they feel to reaching that goal* (Link Worker 4)

### Opportunity

Table 2 outlines eight barriers and facilitators that were identified in relation to opportunity. These were found across two of the theoretical domains: Environmental context and resource, as well as social influences.

#### Environmental context and resource

##### Having appropriate spaces to talk to CYP

For the majority of Link Workers and those involved in the setting up or running of SP for CYP, the need for appropriate spaces to work with CYP was of paramount importance. Participants commented on how some locations may not put an individual at ease and facilitate having open conversations:

*I would love for social prescribing to be in a less formal setting. I work for different [GP] surgeries and I’m either on the phone or in the surgery. And that’s really quite formal. I have to wear like business uniform … If I could turn up to a community centre and see them there, and I was wearing something more casual, like jeans and a nice top, there might be less of that power imbalance that I mentioned* (Link Worker 7)

Locations where Link Workers could meet CYP varied, but shared the feature of being welcoming and friendly. Schools and leisure centres were popular choices, as well as online.

##### Service limitations on who could be worked with

Some Link Workers and those in management and implementation roles outlined that there were limits, often due to contracts and funding, on the populations they were able to work with:

*So a lot of the link workers we work with, they work with parents, they can only work with 18*+*, so it’s often through the parents that they have that contact with the family, and then the child is referred via the family, as opposed to direct contact with the child* (Social Prescribing Co-ordinator 1)

##### Access to adequate clinical supervision

For Link Workers, having appropriate clinical supervision was important. Often, they were working with vulnerable CYP suffering from mental health difficulties. Supervision was, therefore, a structure to make sure they, as well as the CYP, were being properly supported:

*I think absolutely everyone should have supervision in every single role that they have. As someone to complain about the cases that you're seeing, to make sure that you're on the right track, to highlight any new services and resources that you can tap into. Often you need that reassurance that you’re doing everything you can* (Link Worker 7)

Despite this, some Link Workers did not receive any supervision, whilst others felt the supervision that they received was inadequate:

*And in terms of supervision from a manager, again, I feel as if they do check-in on what I’m doing, but I’ve never felt as if they were really giving me that feedback…It’s more just kind of, if you’re ticking the box, that you’ve had that conversation, then that’s good* (Link Worker 6)

Co-ordinators and those involved in service delivery also agreed that supervision was essential, but often ad hoc and not formalised:

*We’re asking them [Link Workers] to do the toughest of jobs, and we’re not giving them any outlet for those emotions that come up, or for the challenges that come up. So absolutely supervision that’s really built in [is essential]* (Social Prescribing Co-ordinator 1)

##### Funds for CYP to engage in activities

Money was something that almost all participants suggested as helping to facilitate SP. For Link Workers, money was required to help CYP access activities which were not free in the community. For those that did not have pots of funding for activity spending, this meant CYP often had restricted or time limited options available:

*I think for a lot of them, it’s about money. So, a lot of them, they’re living in poverty. Like for people who have a lot of money, and stuff like that, then they… that’s not really a problem, is it? So, if somebody who’s quite a well-off family…they’ll just book them in and they’ll go. I think for kids that are from lower income families, it’s not like that, because you’re then having to choose between food, and that, and gas and electric, and that, and you just can’t afford it* (Link Worker 8)

Not knowing where the money was coming from, or who was going to pay for activities often meant Link Workers felt caught in the middle of a broken, bureaucratic system.

##### Tools to facilitate CYP engagement in SP

Around half of the Link Workers outlined that they used tools to help engage CYP in SP. This included aspects such as cards, board games and sports equipment. Both Link Workers, as well as those involved in the setting up or running of SP for CYP, saw such tools as important to make individuals feel comfortable and to facilitate positive relationships:

*We have a little kit, so when somebody starts, Jenga is our go-to guy for an activity. So we have a kit of resources that we would take in. We do use a workbook for the resilience programme, and we have workbooks for other programmes. But we tend to have a selection of games, art and craft materials, drawing, colouring in, and the young people, we play to their strengths. So if we've got a young person who actually likes to kick a ball around or is very lively, we'll go outside and we'll run their session whilst we're playing a little game of football or basketball* (Social Prescribing Organisational Head 3)

#### Social and professional influences

##### Clinical leadership involvement in SP design

Link Workers and those in middle-management roles outlined how clinical leadership influenced how SP was delivered. Clinical leadership included GPs who led primary care networks (PCN)s, as well as commissioners. These individuals often were involved in hiring the Link Workers and setting up the SP pathways. Thus, they determined how referrals could enter the system, and what types of populations Link Workers could engage with:

*So the staff at the surgery have a big impact, especially partnered GPs and practice managers…clinical directors of the PCN as well, based on if they’re willing to sign or if they’re not willing to sign [contracts with SP services], and that will affect, obviously, children’s access to our service* (Social Prescribing Co-ordinator 1)

##### Community assets’ remit to accept or block onward referrals

Community assets and third sector organisations also influenced SP. Link Workers and Co-ordinators spoke about needing to form good relationships with these partners, to be able to refer CYP into them:

*I mean the community organisations we work with, they can influence the process, massively. We need them to be able to accept young people otherwise the system malfunctions* (Link Worker 9)

If organisations were unable to accept referrals, Link Workers then had to work with CYP to find another appropriate activity.

##### Policy and practice groups sway in the direction of SP

Other stakeholders which influenced SP with CYP, revolved around policy and practice organisations, namely, NHS England, The National Academy for Social Prescribing, and the SPYN. Such organisations were seen as pushing the agenda forward around SP with CYP, and often a key source of support for those in management positions wanting to set up SP:

*NHS England, for example, who’ve put a lot of effort into social prescribing recently, they did describe it as an all-age service from the outset. So their expectation was that it should be available to young people* (Social Prescribing Organisational Head 1)

Similarly, the SPYN were discussed as having spearheaded initial guidance on how to work with CYP:

*I’ve look at quite a lot of the stuff that they [SPYN] have produced on how to work with young people* (Social Prescribing Manager 2)

### Motivation

Table 3 outlines thirteen barriers and facilitators that were identified in relation to motivation. These were found across six of the theoretical domains: Beliefs about capabilities, beliefs about consequences, optimism, motivation/goals, reinforcement and emotion.

#### Beliefs about capabilities

##### Feeling confident engaging with CYP

The majority of Link Workers discussed feeling confident engaging with CYP, feeling that they had the necessary skills and experience to carry out their role. Central to this were interpersonal skills, such as building relationships, empathising, listening, and talking:

*I mean I feel like I’m a good listener and I’m good at talking to young people* (Link Worker 9)

##### Feeling less confident engaging with parents and guardians

However, some Link Workers felt less confident working alongside parents and guardians:

*I’m so used to speaking to a service user directly…But I’m not very well versed in how to speak to a parent about their child who I’m supposed to be helping* (Link Worker 5)

In particular, managing and navigating situations where families differed in terms of what SP activities the CYP should pursue was outlined as potentially challenging:

*I would be less confident in terms of the inter-relationship between the parent and the child. So, for example, I think there would quite often be conflict of interest, where the child would say they wanted one thing, and the parent would say they wanted another thing. And I think negotiating that would be quite difficult* (Link Worker 6)

##### Feeling less confident that some clinicians saw a role for SP

For some Link workers, particularly those split across multiple sites, engaging with clinical teams felt disjointed. They spoke about how SP sat outside of traditional clinical systems, and as a result, felt less confident that they were seen as a valid and useful part of the healthcare team:

*But what tends to happen is that social prescribers sit outside of that, and direct, and it’s not really, I guess, partnership working, it's more that everyone’s aware where everybody is in the system, but they’re not genuinely collaborating to get the best outcomes for people.* (Link Worker 3)

This was echoed by other Link Workers who worried about not getting referrals through as they were forgotten about:

*… how to maintain that contact with people, how to be present without being present physically. And I've really struggled with that. I don't know if it is, but for me it feels like that's part of the reason why the numbers of referrals and the flow of referrals aren't as high as I'd like them personally* (Link Worker 11)

##### Feeling confident around implementing and executing SP

For those in management roles, implementing and executing SP pathways was something that they felt confident doing. This included understanding the detail behind SP and taking it through to execution to benefit service users:

*What am I most confident about? I’m quite confident about how we communicate this stuff. I’m quite confident about organising things. I’m fairly confident about detail, and making sure that when we’ve come up with a good idea, or someone’s come up with a good idea, that we actually plan it through in a way that works* (Social Prescribing Organisation Head 1)

#### Beliefs about consequences

##### Social prescribing empowers CYP and helps well-being

All participants thought that SP was beneficial to CYP as it helped empower them. This was often via connecting them with their peers who had similar interests and activities:

*I think it can really be life changing, sometimes lifesaving as well, with how low some of the young people are feeling, then it can really make a difference. Having that support from other people around them, their peers. And just being able to speak to other people, finding new hobbies, new interests can be fantastic for them* (Link Worker 1)

These interactions and shared interests resulted in positive impacts to CYP’s self-esteem and resilience, meaning they were better equipped to face and manage their difficulties:

*[Social prescribing results in] children who know themselves, who are resilient, who are realistic about life and about their own ability to overcome challenges because they’ve got that innate now strength* (Social Prescribing Co-ordinator 1)

##### CYP getting too attached

Around half of Link Workers discussed that without clear boundaries, it was possible that CYP could become too attached or dependant on the support that they were providing:

*Sometimes they [young people] might become very reliant on the person who is helping them, in the way that they struggle to continue on without that person guiding them and giving them advice, or signposting them to relevant services. So it’s really, really important for the social prescriber to not just give advice, but also give them the encouragement to be able to, I guess, handle things on their own* (Link Worker 5)

Link Workers were mindful of possible attachment risks, particularly when they were not far off the age of the CYP, and worried about the distinction between social prescribing support and peer support being blurred.

##### Social prescribing is not a fix for everyone

Link Workers and those involved in the setting up or running of SP for CYP, cautioned against it being seen as a panacea for treating all difficulties. As the evidence base is still in its infancy, it was posited whether certain types of difficulties may be less suited to SP, such as for individuals that do not like making decisions or that are severely shy:

*I guess, would be that some kids are really shy, and they wouldn’t want to do it, because they wouldn’t want to be part of a group, or doing something like that* (Link Worker 9)

#### Optimism

##### Social prescribing helps CYP

The majority of Link Workers and those involved in the setting up or running of SP for CYP were optimistic that it was effective in helping them achieve their goals and improve their mental health:

*Oh very optimistic…within most goals we kind of rate how happy they are with that, with where they are on the scales. And there’s notable differences sort of before and after engaging with a social prescriber* (Link Worker 4)

However, given the novelty of social prescribing with this population, it was acknowledged that more evidence was needed to confirm this, beyond small-scale, anecdotal experiences:

*We believe, but we haven’t proved it yet…I think there’s a lot more potential outcomes for young people than there are for adults in a way, but yet to be proven* (Social Prescribing Organisational Head 1)

##### The system is not working as well as it could be

A few individuals in management positions were less optimistic about the implementation of SP. This was particularly around whether current pathways and systems were working as well as they could:

*…Only 20% of these referrals were actually coming through GPs and link workers anyway. So, I was looking at that other 80%, where are the children? Where are they? And how do we reach them, so that they can access this in the best way, the easiest way they can* (Social Prescribing Co-ordinator 1)

Others outlined how SP needed to be inclusive and that if it was not implemented as such, then certain marginalised communities, who could benefit from SP, may feel alienated and excluded:

*…[If] we inadvertently exclude certain communities from it. So it starts to take off and becomes a thing that only certain sections of the youth population feel is for them…we have to take care to make sure it’s fully inclusive from the start.* (Social Prescribing Head 1)

Thoughts on overcoming these difficulties were to embed Link Workers in locations, where CYP are situated or frequent, such as schools and places of worship.

#### Motivation/goals

##### Giving the CYP new opportunities and experiences

One motivation around SP with CYP, outlined by some Link Workers and Co-ordinators, was being able to provide them with opportunities and experiences that they may not otherwise have:

*We just want young people to have opportunities, really. Like a lot the young people we work with in schools, particularly, are very isolated…we want to just provide opportunities for people, and spend the money as well* (Social Prescribing Co-ordinator 2)

##### Wanting to improving outcomes for CYP

Linked to above, a motivation for all involved was to improve the outcomes of CYP they came into contact with. Whilst the exact goal or outcome the CYP wanted to achieve depended on their specific circumstance, those involved in SP spoke of being strongly motivated to helping them achieve these and see the associated difference it would make:

*I think that I enjoy seeing that change in young people and knowing that I've made a difference to them. And being able to go and make a change in their life* (Link Worker 1)

#### Reinforcement

##### High job satisfaction from helping CYP

The majority of Link Workers and those involved in the setting up or running of SP for CYP, spoke of their role in SP as rewarding and satisfying, as they made a difference to individuals who were struggling:

*You feel good when you help someone, and you want to keep helping other people, and that’s a really big positive* (Link Worker 6)

The results of seeing the positive impact on CYP acted like a catalyst, motivating and spurring those working in the field to go on and help others.

#### Emotion

##### Social prescribing can be draining and stressful

Link Workers discussed how working with CYP who needed mental health support could impact on their own emotions and mental health. In particular, complex and challenging cases could leave Link Workers feeling overwhelmed and spent:

*Social prescribing, it’s tough, it’s a tough job, it really is. It’s so emotionally draining and intense and you have so much to do and so much to think about. So if you’re having a rubbish day or you’ve had a really bad case and you just need to vent or cry, you need to be in an environment that will allow that and not kind of tell you off for getting upset* (Link Worker 9)

Support from others and supervision, where available, were ways that Link Workers were able to decompress these built-up emotions:

*I have access to line management, I can contact the surgery that the child or young person is under to speak to their GP. Which can sometimes take a little while, but is always helpful. I have six-weekly meetings with the safeguarding lead …Oh I attend a [group] every eight weeks* (Link Worker 7)

## Discussion

The aim of this study was to investigate perspectives from the front line around the barriers and facilitators to SP with CYP who have mental health difficulties. This is particularly pertinent given the increased prevalence in mental health disorders in CYP [35, 36], rising health inequalities [6], and global policy shifts to personalised care [37], including SP [2]. The study drew upon the TDF [24], which has been widely used in other settings, but has not been used previously to explore SP for youth mental health.

Interpersonal skills and a knowledge of CYP were identified as facilitators to SP by those seeing service users, as well as those involved in co-ordination and management roles. Interpersonal skills included listening, being encouraging, and having empathy with CYP and were often gained from previous roles and experiences. These align with previous findings exploring GP views on the barriers and facilitators to implementing SP with adults in primary care [28]. This is perhaps not surprising as these skills are seen as important for those working in a caring capacity and are constantly identified as factors influencing person centred care [38], a field which encompasses SP [23]. However, the degree to which this happens in practice should be investigated with many CYP reporting feeling ignored and not included in decisions about care and treatment options [39].

Working with parents/guardians was outlined as a potential barrier by Link Workers when their views differed from the child or young person who they cared for. Previous research has identified considerable discord between CYP and their parents on presenting difficulty at mental health assessment, and ways forward with care and treatment [40]. Similar findings have been identified on how to facilitate treatment decision making with CYP, with clinicians also struggling with managing divergent views between stakeholders [26]. To overcome this, training modules for Link Workers in skills such as negotiation and containment could be beneficial to help equip them to navigate such situations.

The need for effective supervision, as well as clearer policies and procedures around SP were also highlighted as barriers. Many of those working in the field outlined there were not adequate policies for SP and CYP with mental health difficulties. Ineffective training, knowledge and supervision may lead to CYP not benefitting from interventions to their full extent, or in worst case scenarios, could result in harm [41]. As such, procedures, guidelines and quality standards pertaining to Link Worker training and supervision need to be urgently developed and implemented. Research pertaining to some areas, where gaps have been noted, such as managing endings, have already been developed in youth mental health [42] and may provide some good starting points for developing training and resources.

The need for appropriate resources to engage with CYP was highlighted by the majority of those interviewed. This extended to the buildings and locations where Link Workers could meet with CYP to make them feel comfortable and able to build up a trusting partnership. Whilst no locations were explicitly deemed as inappropriate, there was a feeling amongst some that basing services within GP practices and health locations could make it harder to establish a good partnership due to their formality. This echoes other research exploring barriers to person-centred care where both CYP and parents/guardians felt that formal buildings created a hierarchy leading to less trust and openness [27]. Commissioners and those involved in delivering services should consider how best, and where, Link Workers should engage with CYP, including schools and local community hubs [43].

Some of those interviewed queried whether certain CYP characteristics would be suitable for SP. Whilst research is still in its infancy and thus, findings around effectiveness must be treated cautiously, SP has been implemented across different mental health difficulties in both adults [4] and CYP [44], including CYP vulnerable groups [22]. Societal biases exist within SP processes, including Link Workers’ own biases towards certain groups [22] and if not monitored, could led to disenfranchisement, disengagement and unsuccessful outcomes in groups that could derive possible benefits. Currently, there is an overrepresentation of white patients engaging in SP in the UK [45]. This means that either other ethnic groups do not know about these schemes, do not think they are suitable, or think they are, but are choosing not to engage. Research should further investigate this. To tackle health inequalities, pots of funding should be available for CYP to participate in SP activities, as well as the need for assertive outreach with trusted community partners. It should also be noted that SP is not a one-size fits all approach and adaptions to groups who nay struggle to engage should be tested. One example is the London Violence Reduction Programme that has extended the number of Link Worker Sessions available to facilitate relationship building with CYP who may be more sceptical about trusting organisations and agencies [46].

Measures to evaluate SP outcomes for CYP were often mandated by services employing Link Workers. For mental health and well-being, measures clustered around the use of the ONS4 or the SWEMWEBS; however, it was felt by some that these mandated standardised measures were often confusing in terms of concepts or language and did not adequately capture change in the CYP. As a result, goal-based outcomes were often preferred. Using standardised measures is important in allowing for the potential for comparing different models of treatment or care, identifying good practice, and informing quality improvement efforts [47]. However, measures also need to be appropriate for the population in question. For example, previous research has identified that some measures have higher readability ages than the stated age range [48, 49]. Co-developing a common measurement framework, with young person approved and age-appropriate measures, will allow for the better monitoring of SP and can help increase usage from those tasked with administration.

A strength of this study is that it examines a wide range of stakeholder roles regarding SP across the UK that work with CYP and families. This adds breadth to the emerging literature on the topic, outlining some of the commonalities regarding the barriers and facilitators to SP, both from the perspectives of GPs working with adults [28], as well as at four pilot sites exploring SP for CYP [21]. A further strength of this study is the use of the TDF [24]. Rather than asking individuals what they believed to be the barriers and facilitators of SP, a systematic approach examining fourteen domains and underpinned by theory was employed. This may help illustrate a wider range of barriers and facilitators around SP, rather than just the ones that were immediately apparent to participants during the interview. Moreover, a semi-structured approach to the interviews was undertaken, which involved asking patients to elaborate on answers and allowed for deviation from the set TDF questions when relevant. This allowed for a richer narrative to be formed and provided further context to the barriers and facilitators around SP for CYP.

Limitations also exist. Although a mix of roles across the SP process were recruited and interviewed, representation from voluntary and community sector organisations and clinicians were largely missing, as were the barriers and facilitators from CYP and parent/guardian perspectives. This means that we only have part of the picture. Future research should investigate barriers and facilitators around SP for CYP in these other groups. A further limitation is the number of participants that were interviewed as part of this study. Whilst 20 participants are adequate for information sufficiency, it may be that some themes may have become more developed, rich and complex by recruiting more participants. Finally, it should be noted that all those involved in the setting up or running of SP for CYP identified as ‘White or White British’. Thus, experiences around barriers and facilitators from individuals identifying from other ethnic groups is missing. This may be particularly important, given that there is an overrepresentation of white patients engaging in SP [45] as well as those in health management positions [50]. Future research should urgently investigate experiences of those involved in social prescribing from non-white backgrounds to ascertain whether particular barriers can be identified.

## Conclusion

A number of barriers and facilitators have been identified as affecting SP for CYP mental health. Some, such as interpersonal skills, working in informal buildings and locations, and being able to work with both CYP and parents/guardians, have been previously noted, either within the SP field directly, or within the wider field of person-centred care. Novel findings included the lack of policies, procedures and guidelines to implement CYP social prescribing and individuals querying whether SP was appropriate for certain types of difficulties. Understanding all barriers and facilitators could help inform future interventions. For example, the development of measurement frameworks, models of SP for CYP and training modules for Link Workers could help embed SP for CYP. Others, such as whether SP is appropriate for all difficulties, should be carefully evaluated and reported on as the field expands.

### Supplementary Information

Below is the link to the electronic supplementary material.

Supplementary file1 (DOCX 19 KB)
