# Supplementary material for: Barriers and facilitators to social prescribing in child and youth mental health: perspectives from the frontline
Source: Eur Child Adolesc Psychiatry. 2023 Jul 5;33(5):1465–79. doi: 10.1007/s00787-023-02257-x (PMC11098893; doi:10.1007/s00787-023-02257-x)
Supplement: Supplementary file 1 — Supplementary file1 (DOCX 19 KB) [file 787_2023_2257_MOESM1_ESM.docx]

**WHO** is this for? Managers/Directors/Individuals/Senior leaders/Clinicians/Link Workers/Organisations facilitating Social Prescribing

Thank you for taking part in this interview/focus group. We are working on a project which aims to explore the barriers to social prescribing with young people to improve mental health/wellbeing. We have based this questions on a framework for behaviour change, if some of these do not make sense, or do not seem applicable, do let us know and we can clarify or skip it. You are free to stop when you want, or skip any questions. Please tell us if anything is unclear. Do you have any questions before we begin?

[Confidentiality statement] – we may have to share information with others if you tell us something that indicates that you or someone else may be at harm

**Sampling**

This information will help us get a diverse range of views. If you do not wish to provide this information, please tick the box, prefer to not say

Demographic information

1. Age _________________-
2. Gender______________
3. Ethnicity ____________
4. Town/City__________
5. Your role in relation to social prescribing ________________-
6. If you run or organise social prescribing activities, can you tell us more about what types of activities these are ________________-

**Capability**

1. Knowledge

- What do you need to know/be aware of when engaging in (or facilitating) social prescribing with children and young people to improve mental health/wellbeing?
- Do you think you have this awareness/knowledge? Why/why not?
- Are there any policies/guidelines which you follow/are aware of?

1. Skills

- What skills do you need to have to engage in (or facilitate) social prescribing with children and young people to improve mental health/wellbeing?
- Do you think you have these skills? Why/why not/how can this be improved?

1. Memory/attention/decision making

- Can you talk me through the decision making processes when engaging in (or facilitating) social prescribing with children and young people to improve mental health/wellbeing?
- Do these decision-making processes always happen? Why/why not?

1. Behavioural regulation

- How do you monitor your success around engaging in (or facilitating) social prescribing with children and young people to improve mental health/wellbeing??
- Do you get feedback when engaging in (or facilitating) social prescribing with children and young people to improve mental health/wellbeing? From who?

**Opportunity**

1. Environmental context and resources

- What environment are needed (e.g. political, or organisational) to help you engage in (or facilitate) social prescribing with children and young people to improve mental health/wellbeing?
- Do you feel you currently have the right environment to help you engage in (or facilitate) social prescribing with children and young people to improve mental health/wellbeing? Why/Why not?
- What resources are needed to help you engage in (or facilitate) social prescribing with children and young people to improve mental health/wellbeing?
- Do you feel you currently have the right resources to help you engage in (or facilitate) social prescribing with children and young people to improve mental health/wellbeing?? Why/Why not?

1. Social influences

- What people or groups of people (inside or outside your organisation) affect how you engage in (or facilitate) social prescribing with children and young people to improve mental health/wellbeing? Why/how are these affected?

**Motivation**

1. Professional role and identity

[NOTE, if this person is a link worker (or equivalent), miss this question as social prescribing is their role]

- To what extent do you consider engaging in (or facilitating) social prescribing with children and young people to improve mental health/wellbeing to be part of your professional role?
- Why/why not?

1. Beliefs about capabilities

- Are there any steps in engaging in (or facilitating) social prescribing with children and young people to improve mental health/wellbeing that you are particularly confident at?
- Are there any steps in engaging in (or facilitating) social prescribing with children and young people to improve mental health/wellbeing that you are not confident at?
- Why/why not?

1. Beliefs about consequences

- What do you believe are the positive consequences/outcomes of engaging in (or facilitating) social prescribing with children and young people to improve mental health/wellbeing?
- Why?
- What do you believe are the negative consequences/outcomes of engaging in (or facilitating) social prescribing with children and young people to improve mental health/wellbeing?
- Why?

1. Intentions

[NOTE, if this person is a link worker (or equivalent), miss this question as social prescribing is their role]

- To what extent do you intend to engage in (or facilitate) social prescribing with children and young people to improve mental health/wellbeing?
- Why/why not?

1. Reinforcement

- Are you aware in which ways engaging in (or facilitating) social prescribing with children and young people to improve mental health/wellbeing is rewarded?

1. Motivations/Goals

- Do you have any specific motivations or goals engaging in (or facilitating) social prescribing with children and young people to improve mental health/wellbeing?

1. Optimism

- How optimistic are you that engaging in (or facilitating) social prescribing with children and young people improves mental health/wellbeing
- Can you tell me more?

1. Emotions

- To what extent does your emotional state affect whether you are able to engage in (or facilitate) social prescribing with children and young people to improve mental health/wellbeing
- Can you tell me more?
